# Exon Sequencing of* PKD1* Gene in an Iranian Patient with Autosomal-Dominant Polycystic Kidney Disease

**DOI:** 10.6091/ibj.1317.2014

**Published:** 2014-07

**Authors:** Atousa Hafizi, Saeid Reza Khatami, Hamid Galehdari, Gholamreza Shariati, Ali Hossein Saberi, Mohammad Hamid

**Affiliations:** 1 *Dept. of Genetics, Faculty of Science, Shahid Chamran University, Ahvaz, Iran; *; 2 *Narges Medical Genetic Laboratory, Ahvaz, Iran; *; 3 *Dept. of Medical Genetics, Jundishapur University of Medical Science, Ahvaz, Iran; *; 4 *Research Center of Biotechnology, Pasteur Institute of Iran, Tehran, Iran*

**Keywords:** Autosomal dominant polycystic kidney disease (ADPKD), Polycystic kidney diseases (PKD), *PKD1* gene, Iran

## Abstract

**Introduction:** Autosomal dominant polycystic kidney disease (ADPKD) is one of the most common genetic kidney disorders with the incidence of 1 in 1,000 births. ADPKD is genetically heterogeneous with two genes identified: *PKD1* (16p13.3, 46 exons) and *PKD2* (4q21, 15 exons). Eighty five percent of the patients with ADPKD have at least one mutation in the *PKD1* gene. Genetic studies have demonstrated an important allelic variability among patients, but very few data are known about the genetic variation among Iranian populations. **Methods:** In this study, exon direct sequencing of *PKD1* was performed in a seven-year old boy with ADPKD and in his parents. The patient’s father was ADPKD who was affected without any kidney dysfunction, and the patient’s mother was congenitally missing one kidney. **Results:** Molecular genetic testing found a mutation in all three members of this family. It was a missense mutation GTG>ATG at position 3057 in exon 25 of *PKD1*. On the other hand, two novel missense mutations were reported just in the 7-year-old boy: ACA>GCA found in exon 15 at codon 2241 and CAC>AAC found in exon 38 at codon 3710. For checking the pathogenicity of these mutations, exons 15, 25, and 38 of 50 unrelated normal cases were sequenced. **Conclusion:** our findings suggested that GTG>ATG is a polymorphism with high frequency (60%) as well as ACA>GCA and CAC>AAC are polymorphisms with frequencies of 14% and 22%, respectively in the population of Southwest Iran.

## INTRODUCTION

Among all renal diseases, autosomal dominant polycystic kidney disease (ADPKD, OMIM ID: 173900) is the most common inherited kidney disorder [1] that accounts for more than 10% of all cases of end-stage renal diseases (ESRD) [2]. ADPKD is characterized by numerous enlarged fluid-filled epithelial cysts typically in both kidneys and in some cases in other organs. The major extra-renal complications of ADPKD include hepatic cysts, pancreatic cysts, ovarian cysts, prostatic cysts, and cardiac valve disease. In ADPKD, the sensing mechanisms for tubule size seem to be lost; therefore, the cysts are developed and enlarged progressively [3]. PKD causes the progressive cyst formation and ultimately results in renal failure. In ESRD of PKD, many patients depend on either hemodialysis to attenuate renal failure or transplantation; however, no suitable treatment has been developed yet [4]. At present, two causal genes, *PKD1* (MIM 601313) and *PKD2* (MIM 173910) have been identified for ADPKD that are located respectively on chromosome 16 (16p13.3) and chromosome 4 (4q21) [5-7]. *PKD1* gene has 46 exons and encodes a transcript with approximately 14.2 kb in length (NM_000296.3). This gene is extended to 50 kb of the genomic DNA [8] and codes polycystin-1 protein (4302 aa) [9]. The *PKD1* gene is mutated in 85% of all ADPKD cases [8]. On the other hand, *PKD2* encodes a 3-kb open reading frame and has 15 exons which extends to 70 kb genomic areas and produces polycystin-2 protein (968 aa). The *PKD2* gene is mutated in about 15% of ADPKD cases [3]. The exact function of polycystin remains unknown, and the mechanisms of mutations in polycystin led to the pathogenesis of the ADPKD also remain unclear [10]. Polycystin-1 is a receptor protein for cell-cell/matrix interactions that plays crucial roles in the regulation of cell proliferation and apoptosis [11]. Polycystin-2 functions as a transient receptor potential ion channel and regulates the intracellular Ca^2+^ concentration. Polycystin-1 and polycystin-2 interact together to form a functional complex. Their complex acts as a flow-dependent mechanosensor that regulates the differentiated state of tubular epithelial cells [12-14]. Polycystin-1 and polycystin-2 are co-localized in primary cilia and mediate Ca^2+^ signaling as a mechanosensor. Multiple mechanisms have been shown to contribute to PKD, including increased pro-liferation, apoptosis as well as loss of differentiation and polarity. Approximately 50% of individuals with ADPKD have ESRD by age 60 years [15], but the actual age of onset of ESRD depends on the contributed gene. If ADPKD occurs because of *PKD1* mutations, the ESRD may appear as early as the age of 53, but if the *PKD1* is contributed, the age of onset of the ESRD is about 69 [16].

Based on the Human Gene Mutation Database, so far, more than 970 pathogenic mutations have been known for *PKD1* (Table 1). Until now, about 1,920 mutations have been identified in the *PKD1* gene (available through the ADPKD Mutation Database website [http://pkdb.mayo.edu/]). Most of these mutations are point mutations or deletion/insertion mutations that introduce frame shifts and stop codons leading to premature termination. The most likely effect of these types of mutations is loss of polycystin-1 function completely.

Early diagnosis of ADPKD at first is established by ultrasound imaging with age-related cyst number criteria [17], but unfortunately, for younger at-risk individuals and patients with *PKD2* mutations, ultrasonography may be insufficient for providing a definite diagnosis [18]. In these cases, mutation screening, a direct and an effective method, has been established as the applicable method to all cases suspected of ADPKD. This method plays a vital role in the evaluation of related potential kidney donors with doubtful imaging data in individuals with a negative family history, and even in cases of early age of onset of ADPKD. In the present study, we report cases of mutations discovered from the direct mutation screening of all coding exons in the *PKD1* gene in one Iranian family with ADPKD. To verify the pathogenic effect of detected change, 50 unrelated healthy individuals were selected regarding gender, old, and ethnicity for sequence analysis of exon 15, 25, and 38 of the *PKD1* gene. However, very little information is known about how missense mutations might alter the structure of polycystin-1 and mechanical properties we predicted the effects of these mutations on protein polycystin-1 structure.

**Table 1:** The number and type of pathogenic mutations according to Human Gene Mutation Database site for *PKD1* gene

**Primer**	**Sequence**	**Length**	**TM**	**GC%**	**Product (bp)**
Pkd1-15-F	5'-CTGTCCCGGTTCACTCACT-3'	19	58.95	57.89	219
Pkd1-15-R	5'-CTCAGAGCCTGAAAGGCAGT-3'	20	59.68	55.00	
					
Pkd1-25-F	5'-GAGACTGCGACATCCAACCT-3'	20	59.75	55.00	390
Pkd1-25-R	5'-TTCTCAGGATAGAGCCGAGC-3'	20	58.68	55.00	
					
Pkd1-38-F	5'- AGGGTGTGTGCTGCCATTAC -3'	20	60.61	55.00	373
Pkd1-38-R	5'- GGGTCTGGCTGGACTAAAGG -3'	20	59.75	60.00	

TM, melting temperature

**Fig. 1:**
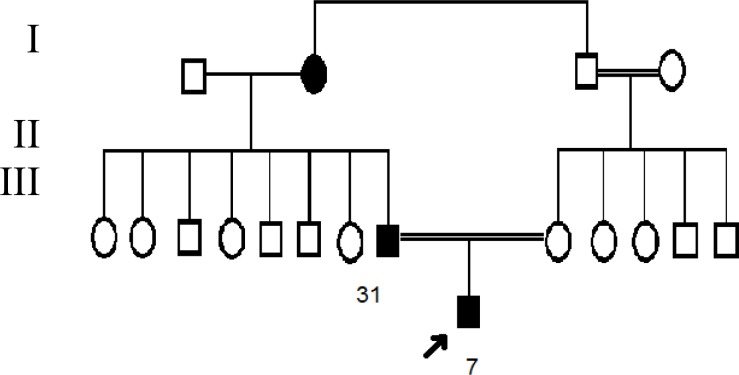
Pedigree of the ADPKD-affected family. The proband is a seven-year-old boy affected by ADPKD (arrow in the pedigree).

## MATERIALS AND METHODS

***Finding the PKD1 mutations in patient’s family.***

***Case*** ***report*** ***. ***The patient was a seven-year-old boy, the only child of consanguineous parents (31 and 35 years old first cousin couple). After obtaining an informed consent, all participants were questioned about their personal medical history, and a family tree was drawn (Fig. 1). On sonography of the abdomen of the proband, the liver was normal in size and normal in echotexture, but contained multiple simple cysts; the largest was 27 × 27 × 25 mm located in the right lobe. On sonography of the kidneys and bladder, both kidneys were normal in position but enlarged in size. Bilateral increased medullary echogenicities due to multiple small parenchymal cysts were seen. There were no stone or hydronephrosis. The lengths of the right and the left kidneys were 112 and 111 mm, respectively, and the parenchymal thickness of both right and left was 11 mm. Serum creatinine level was 0.7 mg/dl. Blood urea nitrogen and urinalysis were normal, and the diagnosis of ADPKD was evoked; therefore, a genetic conformation was asked. In regard to the patient’s parents, the father was affected by ADPKD and had a few cysts in his kidneys with no clinical symptoms, while the mother was healthy with no kidney cysts, but congenitally she had only one kidney.

**Fig. 2:**
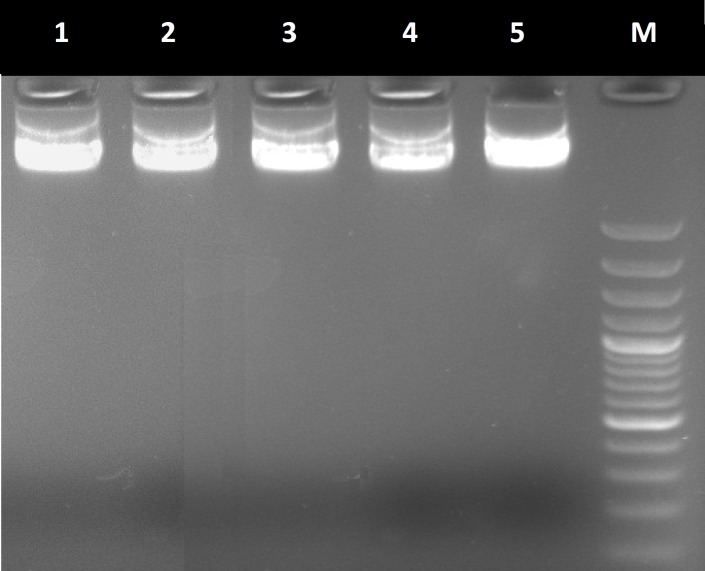
Gel electrophoresis of genomic DNA. Lanes 1 and 2, genomes extracted from normal samples; lanes 3-5, genomes extracted from ADPKD family samples, and M is 1 kb marker

***Sample collection, DNA isolation and polymerase chain reaction. ***Peripheral blood (10 ml) was collected from the patient and his parents in the EDTA tubes. DNA was extracted by the standard salting out protocol, and the quality of the extracted DNA was verified by gel electrophoresis (Fig. 2). Mutations were analyzed based on the PCR-direct sequencing method. *PKD1* gene-speciﬁc PCR primers were synthesized from the region between exons 1 and 46, and a part of the flanking intron sequences were amplified with designed primer pairs (Table 2). The PCR was conducted under the following conditions: 100 ng genomic DNA, 200 μM dNTP, 1.5 mm MgCl, 2.5 units SuperTaq polymerase (Genfanavaran , Iran), and 25 pmol of each primer. For ampliﬁcation of exons and ﬂanking introns, primers were designed by the Primer3 website (http://primer3.ut.ee/). Ampliﬁcation was carried out in 25 μl volumes and 35 cycles: 93°C for 1 min., 60°C for 30 s and 72°C for 45 s. To amplify the full length of each exon, primer positions were chosen on the flanking intron close to the splice sites. The concentration of the primers and Taq polymerase for optimal reaction efﬁciency was determined empirically. The annealing temperature ranged from 60 to 65°C for each primer set. The PCR products were checked by electrophoresis on 1.2% agarose gels and then puriﬁed and directly sequenced.

***Direct sequencing of PKD1 exons of patient’s family.*** Direct sequencing of the exons and the ﬂanking intron sequences were the same as performed using the Big Dye Terminator Cycle Sequencing Ready Reaction Kit (Applied Biosystems) on an ABI Prism 3700 automated genetic analyzer (Applied Biosystems). The same PCR primers were used for sequencing reactions, which were performed with forward and reverse primers. Finally, the sequences were compared to the reported gene sequence using the BLASTN program.

***Sample collection, DNA isolation and Polymerase chain reaction of controls.*** Peripheral blood (5 ml) was collected from 50 ethnicities matched unrelated male controls without any kidney disease in EDTA tubes. DNA was extracted by standard salting out protocol. The PCR was conducted for exons 15, 25, and 38 in the following conditions: 100 ng genomic DNA, 200 μM dNTP, 1.5 mM MgCl, 2.5 units SuperTaq polymerase (Genfanavaran, Iran), and 25 μl pmol each primer. Ampliﬁcation was carried out in 25 volumes and 35 cycles: 93°C for 1 min., 60°C for 30 s, and 72°C for 45 s. The PCR products were qualified by electrophoresis on 1% agarose gel, and then the PCR products were puriﬁed and directly sequenced.

**Table 2:** Primers are listed above for amplification of PKD1 gene that has been designed according reference sequence NT-037887

**Primer **	**Sequence**		**Primer **	**Sequence**
pkd1-E1-1-F	Gatgccagtccctcatcg		pkd1-E20-1-F	tgacagggcagagggttg
pkd1-E1-1-R	Gctccaggcgttccttattt		pkd1-E20-1-R	CAGCAGGGCGTACACCAG
pkd1-E2-1-F	Gtgcagaggaagcccgag		pkd1-E20-2-R	GACCAGGGCCAACGAGTA
pkd1-E2-1-R	gactcaggtgtgggcttcag		pkd1-E20-3-F	GCTCACCGCTAGTGTGCTC
pkd1-E3-1-F	gatgctggcaatgtgtgg		pkd1-E20-3-R	CACAGCAAGCTGTCAGCAG
pkd1-E3-1-R	acagctgagcagcaagagg		pkd1-E20-4-F	CTAGCACGTAACTGCACCCC
pkd1-E4-1-F	ggggcttccatcagcttt		pkd1-E20-4-R	gacagaacggctgaggctac
pkd1-E4-1-R	gagggcagaagggatattgg		pkd1-E21-1-F	gtgtagagaggagggcgtgt
pkd1-E5-1-F	catagacccttcccaccaga		pkd1-E21-1-R	aggagggaggcagaggaa
pkd1-E5-1-R	ctgggaaggacagagctgg		pkd1-E22-1-F	cctccctctacctccctgtc
pkd1-E6-1-F	gagccaggaggagcagaac		pkd1-E22-1-R	CCTGTGTCTGGAATGCCATC
pkd1-E6-1-R	CAGCACATAGCGATGCGAG		pkd1-E22-2-F	AATCCCTTTCCCTTTGGCTA
pkd1-E6-2-F	CTGGGACTTCGGAGACGG		pkd1-E22-2-R	CATAGGAGCCTCTGCACCAG
pkd1-E6-2-R	agggtgtcaacggtcagtgt		pkd1-E22-3-F	TGCTTTCTGTTTCATGGGCT
pkd1-E7-1-F	acactgaccgttgacaccc		pkd1-E22-3-R	gtaacccaggcaatgctgac
pkd1-E7-1-R	tccttcctcctgagactccc		pkd1-E23-1-F	gagactgcgacatccaacct
pkd1-E8-1-F	gtgggaggatggaggagtg		pkd1-E23-1-R	ttctcaggatagagccgagc
pkd1-E8-1-R	ctaaccacagccagcgtctc		pkd1-E24-1-F	gctcggctctatcctgagaa
pkd1-E9-1-F	gtctgttcgtcctggtgtcc		pkd1-E24-1-R	agtgctcacgaggtcattcc
pkd1-E9-1-R	gcaggagggcaggttgtag		pkd1-E25-1-F	cagccatgtttgcatgtcac
pkd1-E10-1-F	ctctccttccctcccctctt		pkd1-E25-1-R	CAGGAGGGAGGTCAGGCT
pkd1-E10-1-R	cagcagacgtgaaagctcag		pkd1-E25-2-F	AGTGTGGCACGACAACAAAG
pkd1-E11-1-F	cagttgggcatctctgacg		pkd1-E25-2-R	AGATGAGGAGAACGCAGCAG
pkd1-E11-1-R	gaggagatgcagggaacaga		pkd1-E25-3-F	TTGACAAGCACATCTGGCTC
pkd1-E12-1-F	atgaccgtgaggacgtgatg		pkd1-E25-3-R	GTGGACGCCTTTCCCTCT
pkd1-E12-1-R	GTTGGTGGGCACGTAGAGG		pkd1-E25-4-F	AGTACTGGGAATGGAGCCTG
pkd1-E12-2-F	AACCTCTCCTGCAGCTTTGA		pkd1-E25-4-R	ATGGGGTCTTGCTATGTTGC
pkd1-E12-2-R	CTGTGTGAGCACCCTGTCTG		pkd1-E25-5-F	CGCCTGTAATCCCAACACTT
pkd1-E12-3-F	GCAGGGAGTCCTAGTGgtga		pkd1-E25-5-R	aaattctatcgtgaaggctctga
pkd1-E12-3-R	ggtcacgccatttctgatg		pkd1-E26-1-F	tctgtcctgtctgctgagga
pkd1-E13-1-F	taagggcagagtcctccaca		pkd1-E26-1-R	cagacacagtgacctgcacc
pkd1-E13-1-R	aagcagagcagaaggcagag		pkd1-E27-1-F	gtgtgacacatcccctggta
pkd1-E14-1-F	ctctgccttctgctctgctt		pkd1-E27-1-R	ctcctctggcaatccccc
pkd1-E14-1-R	tgggacaagagcctggtg		pkd1-E28-1-F	aggttaacatgggcttggct
pkd1-E15-1-F	ctgtcccggttcactcact		pkd1-E28-1-R	ctcacCTTCAGTGGCTCCC
pkd1-E15-1-R	ctcagagcctgaaaggcagt		pkd1-E28-2-F	CTGTGGCTGTCTCAGGGTG
pkd1-E16-1-F	gtgctgctacacctgtgctc		pkd1-E28-2-R	gagacaagagacggaggtgg
pkd1-E16-1-R	GATGTTGTCGCCCGTCTG		pkd1-E29-1-F	caaagccctgctgtcactgt
pkd1-E16-2-F	ACTCAGCGTGGACATGAGC		pkd1-E29-1-R	cctccagttctagcagccac
pkd1-E16-2-R	CGCAGATGCTGGTGAAGTAA		pkd1-E30-1-F	ccatgttccctgggtctct
pkd1-E16-3-F	CGACGGTGACACACAACTTC		pkd1-E30-1-R	gctgctctctcaacaagagga
pkd1-E16-3-R	CCCCAGATCCCACAGGTAG		pkd1-E31-1-F	ccagcaggaaacactcctgt
pkd1-E16-4-F	AGCATCAAGGTCAATGGCTC		pkd1-E31-1-R	CTGTTGTCCAGCCAGTTGTG
pkd1-E16-4-R	TGAGCTGCAGGACATAGACG		pkd1-E31-2-F	TCCTGTGCCGTGTATGACAG
pkd1-E16-5-F	CAATATCATCGTCACGGCTG		pkd1-E31-2-R	gagtgagggtgggctcct
pkd1-E16-5-R	GGTAAATGGCTCGGAGGTCT		pkd1-E32-1-F	agcccaccctcactcctc
pkd1-E16-6-F	GGTGGCAGTGGTGTCGTAT		pkd1-E32-1-R	ACAGCAGCAGGCACACct
pkd1-E16-6-R	GGATCTGAAAATGGACCAGC		pkd1-E33-1-F	CTCTGCTCACCTCGgtacg
pkd1-E16-7-F	CTCAGCCACGTACAACCTCA		pkd1-E33-1-R	ATGTCTTGCCAAAGACGGAC
pkd1-E16-7-R	CACCTGCAGCCCACTCAC		pkd1-E33-2-F	GGCTGCAAGCAGACAGATTT
pkd1-E17-1-F	GTGGCCTACCACTGGGACT		pkd1-E33-2-R	ATCTCGTAGTCCTGGGGCTC
pkd1-E17-1-R	CCCAAATGACACGACAAACA		pkd1-E33-3-F	GCTGGGGGCTGTTATTCTC
pkd1-E17-2-F	GAGTACCGCTGGGAGGTGTA		pkd1-E33-3-R	AGTCGGTCAAACTGGGTGAG
pkd1-E17-2-R	gttctctgggctcatgggt		pkd1-E33-4-F	GGACAAGGTGTGAGCCTGAG
pkd1-E18-1-F	tgctttaaaactggatgggg		pkd1-E33-4-R	TGAAGCCCACAGACAGACAG
pkd1-E18-1-R	AGGGGCTCTTCCTCACTGTT		pkd1-E33-5-F	TTACTTTCTGCCGCTGTCAA
pkd1-E18-2-F	CTTCCAAACCTGCCACAGTT		pkd1-E33-5-R	CAGCAGGTGTTGGGGGAG
pkd1-E18-2-R	GGAGCGTGAGGGTGAGAAC		pkd1-E33-6-F	CTCACTGTGTGTCTCGTGTCAG
pkd1-E19-1-F	TTCCAGCAGGCCAAATAGAC		pkd1-E33-6-R	TACACAGAAGCAGGCACAGC

***Direct sequencing of PKD1 exons of controls.*** Direct sequencing of the exons 15, 25, and 38 and the ﬂanking intron sequences were performed using the Big Dye Terminator Cycle Sequencing Ready Reaction Kit (Applied Biosystems, USA) on an ABI Prism 3700 automated genetic analyzer (Applied Biosystems). The same PCR primers were used for sequence reactions. Sequencing reactions were performed with the forward primers. 

## RESULTS

**Fig. 3:**
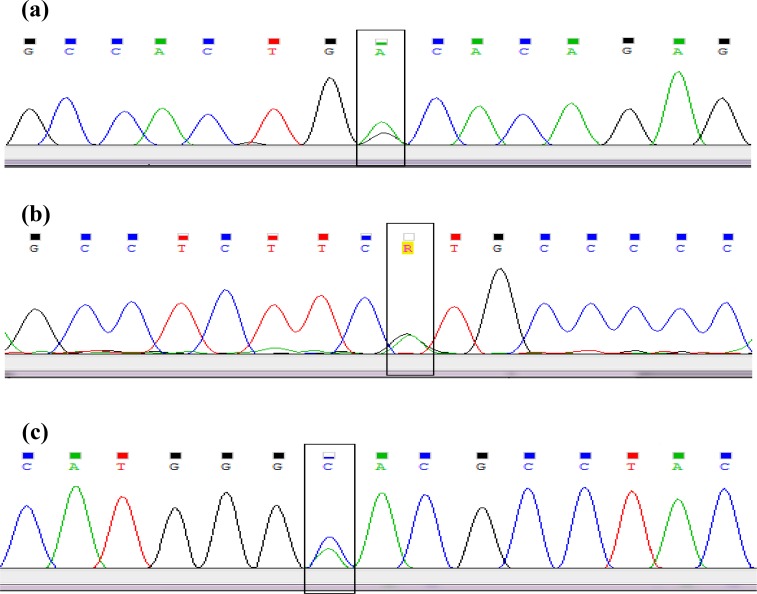
The result of genetic sequencing showing the heterozygous ACA>GCA missense mutation in exon 15 **(a)**, the heterozygous GTG>ATG missense mutation in exon 25 **(b)**, and the heterozygous CAC>AAC missense mutation in exon 38 **(c)** of Patient’s PKD1 gene (sequencing with forward primer).

Direct sequencing analysis of the proband and his parents after comparison with the NCBI Reference sequence of *PKD1* gene demonstrated a heterozygous missense mutation GTG → ATG substitution in the proband and his parent at exon 25, which causes an amino acid exchange of valine to methionine at codon 3057. Moreover, two heterozygous missense mutations were observed just in the patient: ACA→ GCA in exon 15 at codon 2241 that changed threonine to alanine and CAC→ AAC in exon 38 at codon 3710 that changed histidine to asparagine. The results of direct sequencing of *PKD1* exons 15, 25, and 38 in 50 normal individuals demonstrated ACA→ GCA in 9 persons, GTG → ATG in 30 persons, and CAC→ AAC in 11 persons of the normal population. The results of sequencing exons 15, 25, and 38 are given in Figure 3.

## DISCUSSION

**Fig. 4:**
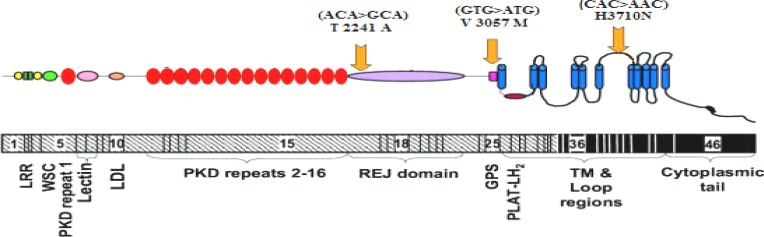
Diagrams of polycystin-1 (upper) and *PKD1 *gene (lower) show domain structures and coding regions for the protein domains, respectively. Three *PKD1 *mutations (above polycystin-1) were identified by our groups.

ADPKD is the most common genetic and inherited kidney disease with an incidence of 1 in 1,000, characterized by an important allelic variety with many variants described [5]. A large number of methods have been used to screen mutations in ADPKD for research and clinical aims, but direct sequencing is still considered as the method of choice [19, 16]. The most frequently reported types of mutations with pathogenic significance are deletions, insertions, splices, frame shift, substitutions, and nonsense mutations [15].* PKD1* gene mutations are considered as an important factor causing ADPKD. However, some observations suggest that genetic susceptibility might contribute to the varied disease phenotype [20]. In about 85% of the patients, mutations occur in *PKD1* gene and in 15% of the cases, *PKD2* has mutations [5]. However, genetic evaluation was performed to have an idea of causal mutation. Method of genotype analysis is variable according to the clinical background and family history; therefore, genetic analysis is not always required in all patients with ADPKD. The preliminary diagnosis is often made by ultrasound, CT scan, and MRI. Young individuals with positive family history are recommended to undertake genetic tests [5]. However, we performed whole gene sequencing of the *PKD1* gene for family members, including affected son, and his mildly affected father, who showed no clinical manifestation of kidney disease. In addition, his mother with known congenital agenesis of right kidney was screened for putative mutations in the *PKD1* gene. As a result, we identified a genetic variant of *PKD1* in ADPKD affected boy and his parents. It was a missense mutation G>A (V3057M) located in exon 25 of the *PKD1* gene. In addition, we found two novel mutations just in affected boy, one of them was a heterozygous A>G missense mutation (ACA>GCA)/N in exon 15 at codon 2241 of *PKD1* gene that changed threonine to alanine in polycystin-1 protein and the other was a heterozygous C>A missense mutation (CAC>AAC)/N in exon 38 at codon 3710 of the *PKD1* gene that changed histidine to asparagine. After that, for verifying the pathogenicity of these three mutations, we performed PCR and direct sequencing of exons 15, 25, and 38 and their flanking regions 50 unrelated controls. All of our controls were healthy males (sex matched with our patient) without any sign of kidney disease, and all of them were in the same ethnicity as the patient’s family from Southwest Iran. Interestingly, the GTG>ATG mutation was observed in 30 unaffected individuals. These results support the idea that this mutation is a polymorphism with an incidence of 60% in the Southwest Iran. As mentioned in the *PKD1* mutation database (http://pkdb. mayo.edu/), the occurrence of the mutation V3057M is rare, but our findings demonstrate that this mutation is very common in the southwest population of Iran. Moreover, our novel mutations (ACA>GCA and CAC>AAC) are polymorphisms with frequencies of 14% and 22% in control samples, respectively. Figure 4 schematically shows the locations of mutations found in the polycystin-1 domains.

**Fig. 5.**
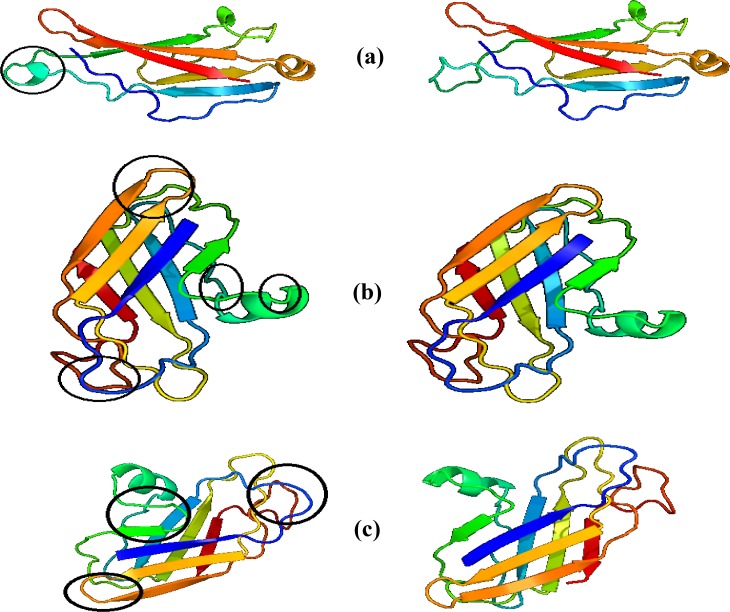
Three dimensional models of PKD1 gene produc. **Right:** predicting the 3D structure of normal REJ domain (a), GPS domain (b), and transmembrane and loop regions (c) of the polycystin-1 protein. **Left:** T2241A mutant REJ domain (a), V3057M mutant GPS domain (b), and H3710N mutant transmembrane and loop region (c) structures. The differences between two domain structures have shown with black circles. Pictures prepared by Phyre2 website

The first mutation ACA>GCA changes threonine to alanine at codon 2241 in REJ (receptor for egg jelly) domain of the polycystin-1 protein. Although the REJ domain is found in the polycystin-1 and in the sperm REJ, the function of this domain is unknown. The domain is 600 amino acids long; therefore, it is probably composed of multiple structural domains. There are six completely conserved cysteine residues that may form disulfide bridges. This region contains tandem PKD-like domains [21, 22]. T2241A makes changes in this domain structure in comparison with the normal domain structure as shown in Figure 5a.

The second mutation GTG>ATG causes the conversion of amino acids valine to methionine at codon 3057 in G-protein-coupled receptor proteolytic site domain (GPS domain) of polycystin-1. This domain is present in latrophilin/CL-1, sea urchin REJ, and polycystin and termed the GPS domain (for GPCR proteolytic site), because it contains a cleavage site in latrophilin [23]. Many surface receptors present thid domain [24]. Currently, there has been no evidence that this domain provides a cleavage site in any of the other receptors. However, the peptide bond that is cleaved in latrophilin is between amino acids leucine and threonine residues that are conserved in some of the other receptors [24]. GPS domains are about 50 residues long and contain either 2 or 4 cysteine residues that are likely to form disulfide bridges. Similar to REJ domain, the exact function of GPS domain is still unknown. V3057M makes some changes in this domain structure in comparison with the normal domain structure as shown in Figure 5b.

The third mutation CAC>AAC changes histidine to asparagine at codon 3710 in transmembrane and loop regions of the polycystin1 protein. Hydropathy plot and computer-assisted analyses identified 9 to 11 transmembrane regions from amino acids 3075 to 4014 with intervening intracellular and extracellular loops [8]. We also tested the effect of H3710N mutation on domain structure (the structure of the polycystin-1 protein domain, Fig. 5c) that differs significantly from wild-type. However, functional prediction of trans-membrane domains is limited by computer algorithms. Therefore, direct biochemical protein purification and crystallization studies are needed to elucidate definitively the structure [25-27].

We conclude that the pathogenicity of some allelic variants is difficult to prove. Nevertheless, it is a first step to future multicenter studies for better description of genetic variants in Iranian ADPKD patients.
